# Safe and effective protocol for discharge 3 days after cardiac surgery

**DOI:** 10.1038/s41598-021-88582-0

**Published:** 2021-04-26

**Authors:** Omar Asdrúbal Vilca Mejia, Gabrielle Barbosa Borgomoni, Nilza Lasta, Mariana Yumi Okada, Mariana Silva Biason Gomes, Mary Lee Norris Nelsen Foz, Helga Priscila Giugno Bischoff, Tatiana Saruhashi, Livia Maria Garcia Melro, Márcio Campos Sampaio, Pedro Gabriel Melo de Barros e Silva, José Carlos Teixeira Garcia, Valter Furlan

**Affiliations:** 1 Hospital Samaritano Paulista, São Paulo, São Paulo Brazil; 2 Department of Cardiovascular Surgery, Instituto do Coração do Hospital das Clínicas da Faculdade de Medicina do Estado de São Paulo (InCor), São Paulo, São Paulo Brazil

**Keywords:** Cardiology, Interventional cardiology, Risk factors

## Abstract

The Enhanced Recovery After Surgery (ERAS) protocol affected traditional cardiac surgery processes and COVID-19 is expected to accelerate its scalability. The aim of this study was to assess the impact of an ERAS-based protocol on the length of hospital stay after cardiac surgery. From January 2019 to June 2020, 664 patients underwent consecutive cardiac surgery at a Latin American center. Here, 46 patients were prepared for a rapid recovery through a multidisciplinary institutional protocol based on the ERAS concept, the “TotalCor protocol”. After the propensity score matching, 46 patients from the entire population were adjusted for 12 variables. Patients operated on the TotalCor protocol had reduced intensive care unit time (*P* < 0.025), postoperative stay (*P* ≤ 0.001) and length of hospital stay (*P* ≤ 0.001). In addition, there were no significant differences in the occurrence of complications and death between the two groups. Of the 10-central metrics of TotalCor protocol, 6 had > 70% adherences. In conclusion, the TotalCor protocol was safe and effective for a 3-day discharge after cardiac surgery. Postoperative atrial fibrillation and renal failure were predictors of postoperative stay > 5 days.

## Introduction

Over time, cardiac surgeries have become more complex, although safer and more effective. Along with technology, advances in teamwork have been responsible for these improvements^1^. However, the length of hospital stay after cardiac surgery has remained constant in recent decades, mainly in underdeveloped countries^2,3^.

In this regard, we highlight the impact of Enhanced Recovery After Surgery (ERAS)^4^, an innovative concept that has disrupted the traditional surgical care through the application of protocols that focus on enhancing patient recovery and generate value in surgeries through implement an optimized perioperative pathway. Evidence on the reduction on hospital length of stay and costs, as well as the reduction of morbimortality, start to gain strength, this time focused on cardiac surgery^5–8^.

For a long time, the care of patients admitted to the hospital to perform cardiac surgery focused only on decreasing surgical aggressiveness or on optimizing isolated anesthetic processes. Minimally invasive surgeries with smaller incisions or without the use of CPB started to be performed to reduce hospitalization times^9^, even so, fast-track protocols for early extubation on adult cardiac surgery^10^ were also not able to reduce the length of hospital stay in isolation. Suggesting that processes within the system need to be connected and synchronized.

Thus, in the current context, the decrease in hospital stay becomes one of the most potential weapons to cardiac surgery, aiming at lower risk of complications, increasing the bed turnover, decreasing the consumption of hospital resources, and helping the sustainability of cardiac surgery programs. The aim of this study was to evaluate whether this protocol based on ERAS was effective and safe in reducing the length of hospital stay after cardiac surgery.

## Material and methods

### Patients and sample

This is an observational and prospective study with a cohort of 664 patients undergoing cardiac surgery at a Brazilian referral center, part of the STS Adult Cardiac Surgery Database^11^, between January 2019 to June 2020. This service line to cardiac surgery patients is engaged in the hospital's quality and safety program^12^, the same accredited by the National Accreditation Organization (ONA) and the Joint Commission. For application of this protocol, the study’s executive committee defined the following criteria.

### Inclusion criteria

All patients scheduled for cardiac surgery were invited to be part of the protocol after acceptance by the surgical teams responsible for the case.

### Exclusion criteria

Patients with STS score > 2, emergency status, use of vasoactive drugs, history of psychiatric disorders, drug addict and/or impaired ambulation.

**Figure 1:**
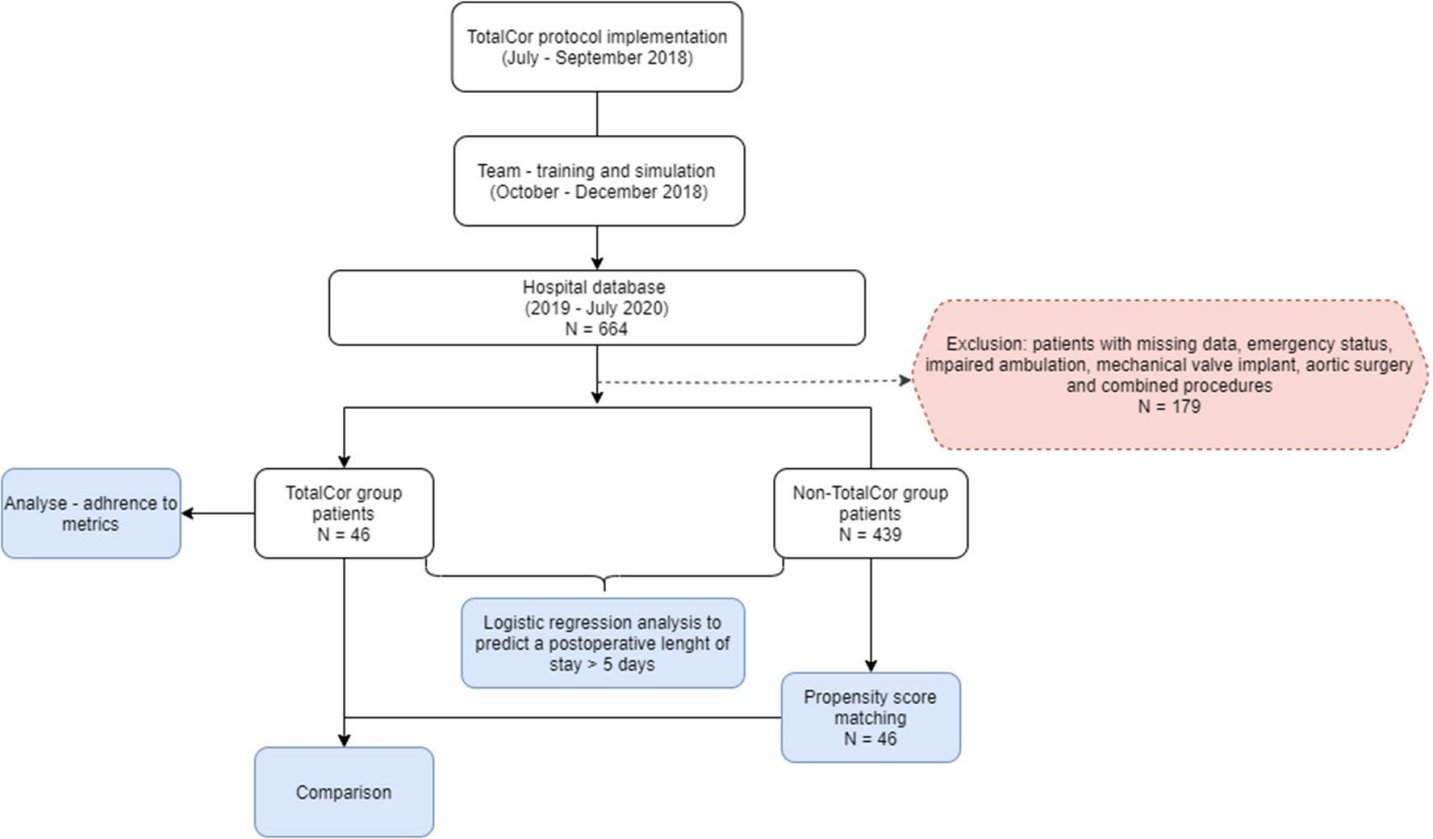
Flowchart of patient selection in the present study.

For the Propensity matching, patients who received mechanical valve prostheses (need for anticoagulation), procedures in the aorta and combined surgeries (coronary artery bypass grafting [CABG] + heart valve surgery, mitral valve + aortic valve surgery, etc.) were excluded. Figure 1 shows the types of analyzes performed to compare outcomes of complications, readmission, and operative mortality.

All patients who agreed to be part of the study were analyzed, so it was an analysis by intention to treat. Operative mortality was defined as (1) all deaths occurring during the hospitalization in which the operation was performed, even if after 30 days; and (2) all deaths occurring after discharge from the hospital, but before the end of the thirtieth postoperative day. The definition of variables and other outcomes follows the STS ACSD version 2.9.

During the study period, there were no changes in the surgical team (surgeons, anesthesiologists, cardiologists, and intensive care physicians) dedicated to this line of care.

### Implementation of TotalCor protocol and team building

After institutional approval, the TotalCor protocol was discussed with the hospital team to implement a multidisciplinary care line. Weekly classes, meetings and training sessions were held to disseminate the concept in the hospital and discuss solutions to the identified obstacles. Implementation, training and simulation of this flow took approximately 6 months.

**Figure 2:**
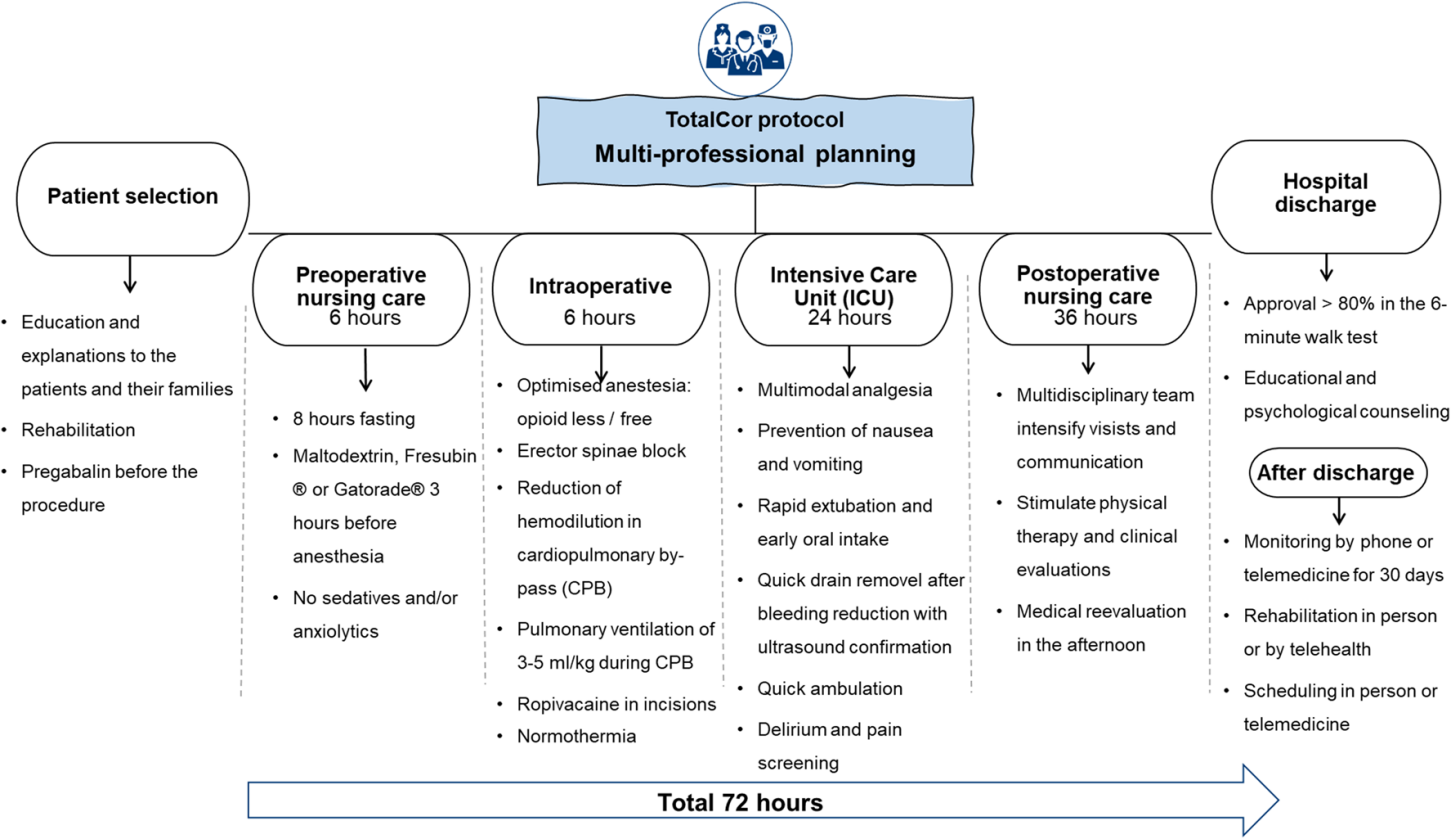
TotalCor protocol flight plan. Visual summary of the TotalCor protocol separated by the hospitalization phases.

Patient data related to the study were collected in a single database. The protocol was coordinated by the Hospital's Cardiac Surgery Quality Program, in multidisciplinary collaboration with anesthesiologists, perfusionists, intensivists, cardiologists, physiotherapists, nutritionists and nursing groups. Thus, in July 2018, this taskforce developed a pragmatic strategy of gradual implementation, which involved the implantation of the TotalCor protocol including the pre-hospitalization phase (elective patients), perioperative care phases in the hospital and follow-up after discharge. Specific measures for the different processes were included in the protocol based on a combination of literature review and institutional experience, and finally approved by the coordinators of the participating groups together with the hospital board. These guidelines consist of metrics for patient care processes (Fig. 2). TotalCor patients were intended to receive these guidelines, unless contraindicated based on existing comorbidities or due to occurrences. Even when any specific intervention was suspended or changed, patients continued to be assessed according to the TotalCor protocol and maintained the ERAS concept. Adherence rates to the process measurements, as well as relevant STS ACS v2.9 variables, were also analyzed. The primary endpoint was the postoperative time after cardiac surgery, as it becomes more suitable for the inclusion of non-elective patients who had to wait for surgery due to preoperative exams, use of anticoagulants and/or antiaggregants, renal protection, etc.

Finally, the TotalCor protocol incorporates Lean Six Sigma concepts^13^ to eliminate waste from traditional surgical flow. Thus, the protocol was designed to be safe for a 3-day perioperative period. In summary, the TotalCor protocol in relation to the routine has minor changes in surgical technique and important changes in anesthesia, perfusion, and care outside the operating room (Supplemental file 1 for protocol details).

### Statistical analysis

In the descriptive analysis, continuous variables were expressed in terms of summary measures (mean, median, standard deviation and quartiles), while categorical variables were expressed as percentages. Bar charts were used to illustrate categorical variables, while boxplots were used to illustrate continuous variables. The propensity score matching (PSM) was used to pair the groups using the nearest neighbor method, available in the "matchit" package of the R software. For the comparison of two groups in continuous variables, the t-test was used for variables that followed the normal distribution (Anderson–Darling test) and for the others, the non-parametric Mann–Whitney and Brunner-Munzel tests were used, respectively, for homogeneous and heterogeneous variables (Bartlett’s test). Fisher's exact test was used for categorical variables. A logistic regression analysis was performed to identify the variables related to a postoperative stay > 5 days after cardiac surgery in the non-TotalCor group. Considering only the variables that were significant in simple regressions, the multiple model was developed. It was validated through the Hosmer–Lemeshow test for the calibration phase and the ROC curve test for the discrimination phase. The level of significance adopted in the tests was 0.05. Two-tailed hypotheses were considered. The software R version 3.6.0 was used to perform all analyzes^14^.

### Ethics and consent

The project was submitted and approved by the Research Ethics Committee of the Hospital Pró-Cardíaco (CAAE: 25123519.6.0000.5533), email address available at: comite.etica@procardiaco.com.br . The free and informed consent was waived by the Ethics Committee due to the analysis of pre-established data logs. We declare that all methods were performed in accordance with relevant guidelines and regulations.

## Results

Of the 664 patients in the registry, 3 were excluded from the TotalCor group and 176 from the non-TotalCor group, resulting in 485 patients (46 TotalCor and 439 non-TotalCor). In general, there were younger patients, with a lower STS risk score and a higher left ventricular ejection fraction (LVEF) in the TotalCor group. These patients also had shorter cardiopulmonary bypass (CPB) time and shorter intensive care unit (ICU), postoperative and total hospital stays, as well as receiving less red blood cell concentrates (Supplemental Material 2). After the PSM, we compared these TotalCor patients with other 46 non-TotalCor patients adjusted for 12 variables: age, gender, functional class, insulin dependence, atrial fibrillation, STS risk score, LVEF, creatinine, type of admission, CPB time, recent acute myocardial infarction (< 21 days) and CABG surgery. Here, we observed that both groups had no significant difference in relation to complications and death, except for ICU stay (days), postoperative hospital stay (days) and length of hospital stay (days) (Supplemental Material 3).

It is worth mentioning that the extubation time in both groups had a median of less than 3 h, an indicator that has encouraged us since the beginning to implement our rapid recovery protocol. The adherence to the goals established for TotalCor patients is shown in Table 1, where the adhesion of 6 metrics was > 70%.

**Table 1:** TotalCor protocol adherence.

Metric	N	%
**10 central measures in the TotalCor patients N = 46**		
1. Preoperative walk test	26	57%
2. Abbreviated fasting	41	89%
3. No preoperative use of sedatives/anxiolytics	39	85%
4. Anesthesia with less opioid/opioid-free	38	83%
5. Erector spinae plane block	34	74%
6. Extubation ≤ 2 h	18	39%
7. ICU time ≤ 24 h	16	35%
8. Afternoon medical re-evaluation	46	100%
9. Postoperative walk test	26	57%
10. Postoperative stay ≤ 5 days	40	87%

A regression analysis was performed to identify the predictors of a postoperative stay > 5 days after cardiac surgery in non-TotalCor patients who survived. Using the variables that were significant in the simple regressions, the multiple model was built (Table 2).

**Table 2:** Multiple regression model to predict postoperative stay > 5 days after cardiac surgery.

Explanatory variable	Coefficient	Standard error coefficient	OR	95% CI	*P* value
Female gender	0.71	0.23	2.03	1.29–3.21	0.002^a^
Preoperative atrial fibrillation	0.97	0.47	2.64	1.04–6.66	0.040^a^
Urgency procedure	0.54	0.22	2	1.10–2.66	0.017^a^
STS risk score	0.38	0.12	1.47	1.15–1.87	0.002^a^
Postoperative atrial fibrillation	1.64	0.36	5.16	2.57–10.34	< 0.001^a^
Renal failure	2.44	0.77	11.45	2.53–51.90	< 0.002^a^

^a^ *P* value < 0.05; CI: confidence interval.

**Figure 3:**
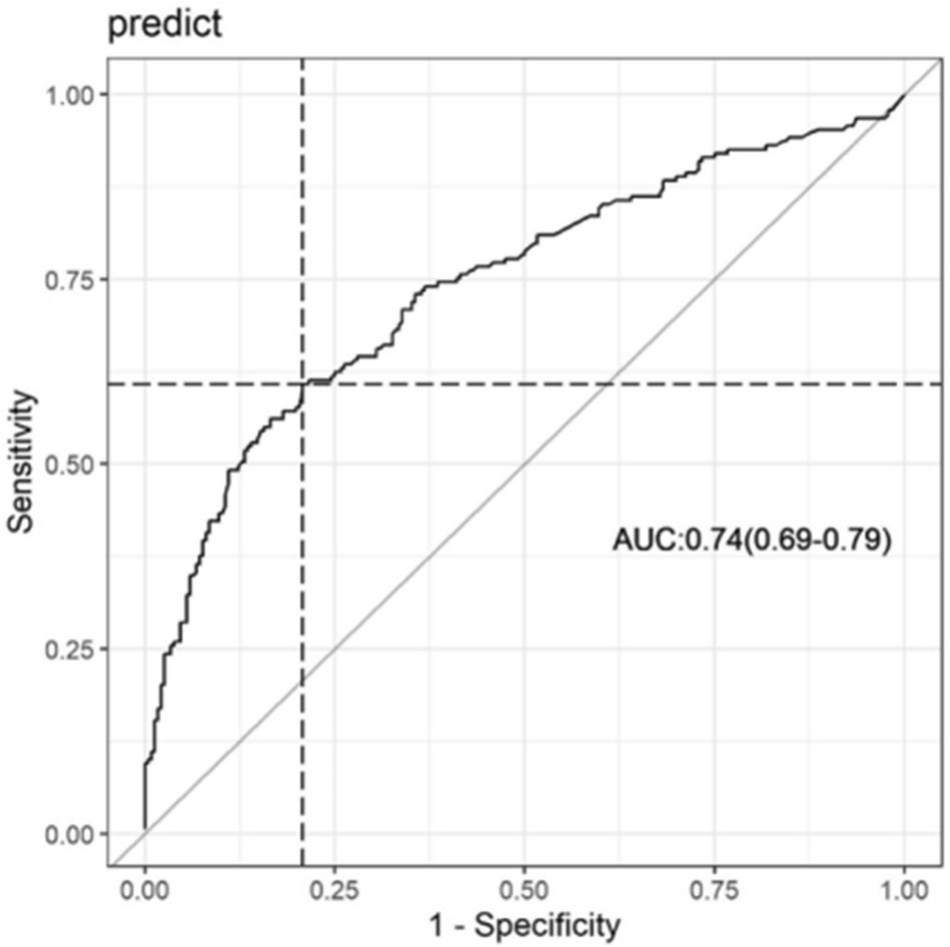
ROC curve of the model to predict postoperative stay > 5 days after cardiac surgery.

To test the model’s performance, the Hosmer–Lemeshow test (*P* = 0.285) showed that the model was well calibrated. Moreover, C statistics (0.74, 95% CI 0.69–0.79) revealed that the multiple model is appropriate to predict postoperative stay > 5 days after cardiac surgery (Fig. 3).

## Discussion

As far as we know, this is the first study using a protocol based on ERACS in Latin America. The TotalCor protocol was safe and effective for hospital discharge 3 days after cardiac surgery. The success achieved in reducing the length of hospital stay is similar to that presented by Chris Malaisrie at STS 2020^15^. Therefore, these findings become an opportunity to redesign the traditional flow of cardiac surgery in the world.

The measures proposed by the enhanced recovery protocols are innovative, break paradigms and directly confront pre-established measures^3^. For this reason, they are being applied slowly and gradually despite evidence of their effectiveness^3,4^.

At first, the ERAS protocol was applied only in colorectal surgeries, generating a considerable volume of scientific evidence within the context of this specialty. With the encouragement of excellent results, other medical specialties started to become interested and to adopt the ERAS concept; with cardiac surgeries, it was not different. Since 2017, professionals from different fields came together to establish the guideline of “Enhanced Recovery After Cardiac Surgery” (ERACS)^1,5–7,16–19^. To add knowledge to the scientific community, we decided to share the results of the TotalCor protocol at Hospital Samaritano Paulista, from São Paulo, Brazil.

Teamwork was essential for the successful implementation of the protocol and can represent a challenge for some institutions^20^. Implementation must be cautious since habits and paradigms are difficult to change^21^. For this, our team took held classes/meetings and training to disseminate the concept and discuss solutions about possible obstacles, and after implementation, our group continues to meet weekly to discuss the process, once the communication between professionals in the different phases of hospitalization is the main factor for the “key” to success.

The average length of postoperative hospital stays after isolated primary elective CABG in one of the reference hospitals in our region is 8.5 days^22^. Therefore, this protocol would have the potential to rotate the bed almost 3 times faster. In the new era, models like this could help maintain essential surgeries, and reducing the consumption of resources for patients hospitalized^23^. We believe that the implementation of an enhanced recovery protocol would be necessary to resume elective cardiac surgeries, as suggested by other surgical specialties^24,25^.

In the 92 patients analyzed in the propensity score, besides the significant reduction in postoperative time, there was a decrease in the appearance of atrial fibrillation from 8.7% in the non-TotalCor group to 4.4% in the TotalCor group, however, although a reduction > 2 times, this was not significant (*P* = 0.159). Perhaps the sample size was not enough to show the results achieved by Malaisrie in Chicago^15^ and by Fleming in London^17^. It should be noted that, although not significant, there was also a decrease in the number of hospital readmissions from 7.1 to 2.2% (*P* = 0.344) in the TotalCor group. For analysis of the metrics, 10 central processes were chosen to assess whether adherence to them was the reason for the success in the application of the protocol, and it was found that there was an adherence greater than 80% in the abbreviation of fasting, elimination of the preoperative use of anxiolytics/sedatives, and application of opioid less/opioid-free concept and postoperative ≤ 5 days. It is worth mentioning that all patients in the TotalCor group underwent medical reevaluations in the afternoon, which, together with the standardization of multidisciplinary practices, must have influenced the preparation and recovery of patients^26,27^.

Goeddel^28^ showed that early extubation improves results; differently, Kandasamy^29^ reported that, even though early extubation offers an advantage over accelerated recovery and shorter ICU and hospital length of stay, it showed no significant difference in patient results. In our analysis, the median extubation time in both groups was less than 3 h and had no significant difference (*P* = 0.477). However, this metric did not affect the reduction in ICU time, postoperative stay and length of hospital stay in the TotalCor group. Here, we found that 91% of patients were extubated within 6 h and 39% within 2 h. As the metric used for this flow was up to 2 h, it becomes an opportunity to improve and revise our flow.

Analyzing the predictors of a postoperative stay > 5 days after cardiac surgery, there are some characteristics of the patients that would help to weigh in the planning of a specific hospitalization. The preoperative and non-modifiable variables were female gender, presence of atrial fibrillation, urgency surgery and an increased STS risk score. Here, perhaps the urgency status and STS value could be improved. However, in practice, it is difficult to find time for clinical optimization, hospital discharge and elective readmission for surgery. Finally, the two outcomes related to a postoperative stay > 5 days were atrial fibrillation and renal failure, which could be mitigated through risk identification and prevention strategies and, in the case of atrial fibrillation, we also have the option of concomitant treatment during the treatment of underlying heart disease, as recently supported by the European guideline^30^.

## Limitations

1. The study is unicentric and coming from a hospital that reports to the STS Cardiac Surgery since 2011. Therefore, is likely that the results may be difficult to be achieved by other centers in other regions. However, the protocol is objective, clear and includes central processes in the journey of the patient who is hospitalized for cardiac surgery. We believe that adherence to the metrics will bring significant results regardless of the characteristics of the hospital. 2. Due to the current COVID-19 pandemic, our rate of inclusion of patients in the protocol has dropped to an average of 2 to 3 patients per month. 3. This was not a randomized study. However, a PSM analysis was performed from a structured, audited, and validated database for cardiac surgery. 4. The group of patients enrolled in the TotalCor Protocol was less than 10%, which limits its impact on the system. To improve this, and after the initial results, we obtained support from the executive management of the hospital so that, from July 2020, all patients with cardiac surgery schedule will be approached within the ERAS concept. Currently, the majority, and not the minority, of patients receive shortening of fasting, modular anesthesia with spinal erector block, as well as intensified physiotherapy protocols. To facilitate the advancement of this culture of patient preparation and optimization, the KAMAY health app is being built with the purpose of engaging patients and hospital staff to improve adherence to metrics.

This new bundled approach to perioperative care is based on the philosophy that patients do better when emotional and physiological stresses are minimized during surgery. The goal of this concept is to return patients to normal functional status as soon as possible. For this, the protocol involves a methodical shift in culture, sustaining a meaningful organizational change, and pivoting to a patient-centered care system. Herein we detail the crucial team building, education, planning, and the processes needed to develop and maintain a successful program.

In conclusion, the TotalCor protocol based on ERAS concept significantly reduced the postoperative hospital stay after cardiac surgery in a safe and effective way. Improvements in team communication through applications, as well as in interaction with patients and family members, should be introduced to increase adherence to the protocol, as well as to continuously improve results.

Further studies should be conducted to balance the advantages and disadvantages of the TotalCor protocol, as well as its sustainability and scalability.

## Supplementary Information

Supplementary Information.
