# Supplementary material for: Safe and effective protocol for discharge 3 days after cardiac surgery
Source: Sci Rep. 2021 Apr 26;11:8979. doi: 10.1038/s41598-021-88582-0 (PMC8076282; doi:10.1038/s41598-021-88582-0)
Supplement: Supplementary file 1 — Supplementary Information. [file 41598_2021_88582_MOESM1_ESM.docx]

**Safe and effective protocol for discharge 3 days after cardiac surgery**

Omar Asdrúbal Vilca Mejia^1,2,^*, Gabrielle Barbosa Borgomoni^1,2^, Nilza Lasta^1^, Mariana Yumi Okada¹, Mariana Silva Biason Gomes^1^, Mary Lee Norris Nelsen Foz^1^, Helga Priscila Giugno Bischoff^1^, Tatiana Saruhashi¹, Livia Maria Garcia Melro¹, Márcio Campos Sampaio¹, Pedro Gabriel Melo de Barros e Silva¹, José Carlos Teixeira Garcia¹, Valter Furlan¹.

¹ Hospital Samaritano Paulista, São Paulo, São Paulo, Brazil.

^2^ Department of Cardiovascular Surgery, Instituto do Coração do Hospital das Clínicas da Faculdade de Medicina do Estado de São Paulo (InCor), São Paulo, São Paulo, Brazil.

***** Correspondence to omar.mejia@incor.usp.br

**Supplemental material 1**

**TotalCor protocol**

1. **Patients selection (elective and urgent patients)**: patients and family members were informed about the protocol and questions were answered before surgery. This meeting with the hospital group would help to reduce perioperative apprehension and discomfort related to misunderstandings. The consent form was obtained after a wide discussion between surgeon, nurse, psychologist, and physiotherapist. Preoperative rehabilitation was offered to elective patients in the protocol.
2. **Hospitalization phase before surgery:** elective patients was scheduled to be admitted to the hospital if glycated hemoglobin was optimized (hemoglobin A1c <8%). In all patients, after eight hours of fasting, maltodextrin/Fresubin or Gatorade was administered 3 hours before anesthesia induction. Sedatives or anxiolytics were not used. Pregabalin were administered before the procedure until the day of the surgery (maximum 5 days).
3. **Intraoperative:** anesthesia was performed under the opioid less or opioid-free concept to improve fast recovery (when necessary, low doses of fentanyl or sufentanil could be administered). Anesthetic induction: multimodal analgesia with dipyrone 30 mg/kg, lidocaine 1.5 mg/kg, magnesium sulphate increasing up to 40 mg/kg and rocuronium 1.2 mg/kg. Patients received erector spinae plane block and the management of anesthesia was modular with ketamine (0.2-0.5 mg/kg), propofol (continue infusion from TCI mode induction, monitored by the bi-spectral index), dexmedetomidine (0.5 a 1.0 mcg/kg for 10 minutes when necessary and maintenance 0.2 a 0.7 mcg/kg/hour) and sevoflurane 0.6 to 1%. In cardiopulmonary bypass (CPB), the perfusionist reduced hemodilution during the procedure, filled the circuit with a pH 7.4 balanced electrolyte solution (prime <1200 ml) and used del Nido cardioplegia. The procedure was performed in normothermia. Dissection of the mammary artery was performed with closed pleura maintaining pulmonary ventilation of 3-5 ml/kg during CPB. The surgeries were performed in normothermia. After CPB, dipyrone and ketorolac were administered to decrease postoperative pain. At the end of the procedure, infiltration with ropivacaine was done in the surgical incisions (0.375% 40 ml). Goal: Patient leaving the room with zero-balance of fluids and body temperature in normothermia (36-37.9ºC).
4. **Intensive care unit (ICU):** early extubation (≤2 hours), rapid mobilization (sitting regularly in a chair after a few hours after surgery and walking around the ICU, early oral intake, early drain removal (it can be safely removed after the drainage becomes macroscopically serous and with a confirmatory ultrasound image). After extubation, paracetamol and codeine can be administered regularly, as well as morphine sulfate, only if necessary. Intravenous ondansetron was used regularly for the first 48 hours after surgery to prevention of nausea and vomiting. Multimodal analgesia is always used to preserve the use of opioids. Scales for periodic pain and delirium assessment were applied. With the patient awake and stable, motor physiotherapy was started, sitting on the bed, in the armchair and, if tolerable, walking with the physiotherapy team. In the sequence, if everything happened as planned; the patient was discharged from the ICU. It is important to highlight the importance of family engagement in this phase of care.
5. **Post-operative nursing care:** respiratory and motor physiotherapy was intensified with multidisciplinary evaluations and an afternoon medical visit focusing on the patient's rapid recovery.
6. **Hospital discharge:** the discharge criterion required 80% approval in the 6-minute walk test with educational and psychological counseling regarding recovery.
7. **After discharge:** patients are monitored by phone or telemedicine for 30 days, scheduling in person or telemedicine consultation within 48 hours and rehabilitation in person or by telehealth. It is important to highlight that the patient and the medical team had free contact access during the postoperative period up to 30 days, thus, the patient was assisted by the medical team, even by telemedicine/telehealth or phone calls as well as the patient had his doubts and wishes resolved by the team whenever necessary.

**Supplemental material 2**

**TotalCor versus non-TotalCor patient’s database**

| **Clinical characteristics** | **TotalCor patients (N=46)** | | | **Non-TotalCor patients (N=439)** | | | **P value** |
| --- | --- | --- | --- | --- | --- | --- | --- |
|  | **N** | **%** | | **N** | **%** | |  |
| **Preoperative - Intraoperative** | | | | | | | |
| **Age, median (IQR)** | 58.3 (53.3-63.8) | | | 62.8 (53.8-70.2) | | | 0.016^c^ |
| **Male gender** | 30 | 65.2% | | 296 | 67.4% | | 0.744 |
| **Insulin dependent** | 2 | 4.4% | | 49 | 11.2% | | 0.207^c^ |
| **Creatinine, median (IQR)** | 0.9 (0.8-1.1) | | | 1.0 (0.8-1.2) | | | 0.123 |
| **Recent myocardial infarction**  **(< 21 days)** | 10 | 21.7% | | 111 | 25.3% | | 0.721 |
| **NYHA class** |  |  | |  |  | | 0.325 |
| Class I | 39 | 84.8% | | 327 | 74.5% | |  |
| Class II | 6 | 13.0% | | 63 | 14.4% | |  |
| Class III | 1 | 2.2% | | 43 | 9.8% | |  |
| Class IV | 0 | 0% | | 6 | 1.4% | |  |
| **LVEF, median (IQR)** | 63.5 (58.5-66.0) | | | 59.0 (50.5-65.0) | | | 0.010^c^ |
| **STS, mean, ±SD** | 0.40, ±0.34 | | | 1.18, ±1.66 | | | <0.001^c^ |
| **Surgery status** |  | |  |  | |  | 0.117 |
| Elective | 32 | | 69.6% | 251 | | 57.2% |  |
| Urgency | 14 | | 30.4% | 188 | | 42.8% |  |
| **CPB (min), median (IQR)** | 52.5 (36.3-76.0) | | | 70.0 (50.0-88.0) | | | 0.002^c^ |
| **Post-operative** | | | | | | | |
| **ICU time (days) median, ±SD** | 1.0, ± 0.65 | | | 2.0, ±2.79 | | | <0.001^c^ |
| **Extubation time (hours), median (IQR)^a^** | 2.8 (1.6-3.9) | | | 3.0 (2.1-4.3) | | | 0.176 |
| **Need for transfusion of blood components** | 3 | | 6.5% | 96 | | 21.95% | 0.012 ^c^ |
| **Surgical-site infection** | 0 | | 0% | 8 | | 1.8% | 1.000 |
| **Sepsis** | 0 | | 0% | 2 | | 0.5% | 1.000 |
| **Delirium** | 0 | | 0% | 9 | | 2.1% | 1.000 |
| **Stroke** | 1 | | 2.2% | 21 | | 4.8% | 0.710 |
| **Renal failure** | 1 | | 2.2% | 27 | | 6.2% | 0.502 |
| **Atrial fibrillation** | 2 | | 4.4% | 59 | | 13.4% | 0.099 |
| **Reoperation** | 0 | | 0% | 5 | | 1.1% | 1.000 |
| **Postoperative stay (days) median, ±SD (IQR)** | 3.0, ±1.20 (3.0-4.0) | | | 5.0, ±6.61(4.0-7.0) | | | <0.001^c^ |
| **Postoperative stay** |  | |  |  | |  | <0.001 ^c^ |
| ≤ 5 days | 44 | | 95.7% | 250 | | 57.0% |  |
| > 5 days | 2 | | 4.4% | 189 | | 43.1% |  |
| **Length of hospital stay (days) median, ±SD (IQR)** | 4.0, ± 3.33 (3.0-7.0) | | | 7.0, ± 7.50 (5.0-11.0) | | | <0.001^c^ |
| **30-days Readmission** | 1 | | 2.2% | 27 | | 6.2% | 0.341 |
| **Death^b^** | 0 | | 0% | 22 | | 5.0% | 0.251 |

^a^ Counting of the time by the departure from the operating room to orotracheal extubation; ^b^ Observed mortality including peak of COVID-19 pandemic period in Brazil; ^c^ *P* value <0.05; IQR: interquartile range 0.25% - 0.75%; LVEF: left ventricular ejection fraction; CPB: cardiopulmonary bypass.

**Supplemental material 3**

**Clinical characteristics between TotalCor and Non-TotalCor patients (Propensity Score Matching).**

| **Clinical characteristics** | **TotalCor patients (N=46)** | | | **Non-TotalCor patients (N=46)** | | ***P* value** |
| --- | --- | --- | --- | --- | --- | --- |
|  | **N** | **%** | | **N** | **%** |  |
| **Preoperative - Intraoperative** | | | | | | |
| **Age mean, ±SD** | 56.79, ± 11.96 | | | 58.45, ± 8.28 | | 0.830 |
| **Male gender** | 30 | 65.2% | 34 | | 73.9% | 0.497 |
| **STS risk score mean, ±SD** | 0.40, ± 0.34 | | | 0.45, ±0.35 | | 0.554 |
| **LVEF mean, ±SD** | 60.93, ±9.61 | | | 62.80, ±7.45 | | 0.584 |
| **Creatinine mean, ±SD** | 0.97, ± 0.21 | | | 0.99, ± 0.28 | | 1.000 |
| **Recent myocardial infarction (< 21 days)** | 10 | 21.7% | | 11 | 23.9% | 1.000 |
| **NYHA class** |  |  |  | |  | 0.315 |
| Class I | 39 | 84.8% | 43 | | 93.5% |  |
| Class II | 6 | 13.0% | 3 | | 6.5% |  |
| Class III | 1 | 2.2% | 0 | | 0% |  |
| Class IV | 0 | 0% | 0 | | 0% |  |
| **Atrial Fibrillation** | 0 | 0% | 0 | | 0% | 1.000 |
| **Insulin dependent** | 3 | 6.5% | 2 | | 4.4% | 1.000 |
| **Surgery status** |  |  |  | |  | 0.659 |
| Elective | 32 | 69.6% | 29 | | 63% |  |
| Urgency | 14 | 30.4 | 17 | | 37% |  |
| **CABG surgery** | 35 | 76.1% | 40 | | 87% | 0.188 |
| **CPB (min) median, ±SD** | 52.5, ± 31.03 | | | 54.5, ± 20.81 | | 0.607 |
| **Post-operative** | | | | | | |
| **ICU time (days) median, ±SD** | 1.0, ± 0.65 | | | 2.0, ± 5.45 | | 0.025^b^ |
| **Extubation time (hours (hours) median, ±SD^a^** | 2.8, ± 21.29 | | | 2.9, ± 1.87 | | 0.477 |
| **Need for transfusion of blood components** | 3 | 6.5% | | 8 | 17.4% | 0.197 |
| **Surgical-site infection** | 0 | 0% | | 1 | 2.2% | 1.000 |
| **Sepsis** | 0 | 0% | | 0 | 0% | 1.000 |
| **Delirium** | 0 | 0% | | 0 | 0% | 1.000 |
| **Stroke** | 1 | 2.2% | | 1 | 2.2% | 1.000 |
| **Atrial fibrillation** | 2 | 4.4% | | 4 | 8.7% | 0.677 |
| **Renal failure** | 1 | 2.2% | | 1 | 2.2% | 1.000 |
| **Reoperation for bleeding** | 0 | 0% | | 1 | 2.2% | 1.000 |
| **Postoperative stay (days) median, ±SD (IQR)** | 3.0, ± 1.20 (3.0-4.0) | | | 5.0, ± 12.70 (4.0-6.0) | | <0.001^b^ |
| **Postoperative stay** |  |  | |  |  | <0.001^b^ |
| ≤ 5 days | 44 | 95.7% | | 29 | 63% |  |
| > 5 days | 2 | 4.4% | | 17 | 37% |  |
| **Length of hospital stay (days) median, ±SD (IQR)** | 4.0, ± 3.33 (3.0-7.0) | | | 7.0, ± 11.96 (4.0-9.0) | | <0.001 ^b^ |
| **30-days readmission** | 1 | 2.2% | | 3 | 7.1% | 0.344 |
| **Death^c^** | 0 | 0% | | 1 | 2.2% | 1.000 |

^a^ Counting of the time by the departure of the operating room to orotracheal extubation ^b^ *P* value <0.05; **^c^** Observed mortality including peak pandemic period in Brazil; IQR: interquartile range 0.25%-0.75%; SD: standard deviation.

**Supplemental material 4**

**Cause of Readmission in patients operated on TotalCor and Non-TotalCor group.**

| **Cause of Readmission** | **Non-TotalCor group (N=439)** | **TotalCor group (N= 46)** |
| --- | --- | --- |
| Surgical site infection | 5 | 0 |
| Pericardial effusion + drainage | 2 | 0 |
| Pleural effusion + drainage | 3 | 1 |
| Heart failure | 4 | 0 |
| Pneumonia | 1 | 0 |
| Cardiogenic shock + cardiorespiratory arrest | 1 | 0 |
| Sepsis + acute renal injury | 1 | 0 |
| DSWI + Urinary tract infection | 1 | 0 |
| Right ventricular dysfunction | 1 | 0 |
| Bacteremia | 1 | 0 |
| Bradycardia | 1 | 0 |
| DSWI with re-approach | 1 | 0 |
| DSWI without re-approach | 1 | 0 |
| Pulmonary thromboembolism | 1 | 0 |
| Atrial Flutter | 1 | 0 |
| COVID-19 + Pleural effusion | 1 | 0 |
| Acute pancreatitis | 1 | 0 |

*DSWI = Deep Sternal Wound Infection
